# Effects of Taxifolin in Spontaneously Hypertensive Rats with a Focus on Erythrocyte Quality

**DOI:** 10.3390/life12122045

**Published:** 2022-12-07

**Authors:** Tomas Jasenovec, Dominika Radosinska, Marta Kollarova, Peter Balis, Stefan Zorad, Norbert Vrbjar, Iveta Bernatova, Sona Cacanyiova, Lubomira Tothova, Jana Radosinska

**Affiliations:** 1 Institute of Physiology, Faculty of Medicine, Comenius University in Bratislava, 813 72 Bratislava, Slovakia; 2 Institute of Immunology, Faculty of Medicine, Comenius University in Bratislava, 811 08 Bratislava, Slovakia; 3 Centre of Experimental Medicine, Slovak Academy of Sciences, 841 04 Bratislava, Slovakia; 4 Institute of Experimental Endocrinology, Biomedical Research Center, Slovak Academy of Sciences, 845 05 Bratislava, Slovakia; 5 Institute of Molecular Biomedicine, Faculty of Medicine, Comenius University in Bratislava, 811 08 Bratislava, Slovakia

**Keywords:** taxifolin, erythrocytes, hypertension, renin–angiotensin system, oxidative stress, nitric oxide, Na,K-ATPase, ACE2, osmotic resistance

## Abstract

Oxidative stress and multiple erythrocyte abnormalities have been observed in hypertension. We focused on the effects of angiotensin-converting enzyme 2 (ACE2) inhibition by MLN-4760 inhibitor on angiotensin peptides, oxidative stress parameters, and selected erythrocyte quality markers in spontaneously hypertensive rats (SHR). We also investigated the potential effects of polyphenolic antioxidant taxifolin when applied in vivo and in vitro following its incubation with erythrocytes. SHRs were divided into four groups: control, taxifolin-treated, MLN-4760-treated, and MLN-4760 with taxifolin. MLN-4760 administration increased the blood pressure rise independent of taxifolin treatment, whereas taxifolin decreased it in control SHRs. Body weight gain was also higher in ACE2-inhibited animals and normalized after taxifolin treatment. However, taxifolin did not induce any change in angiotensin peptide concentrations nor a clear antioxidant effect. We documented an increase in Na,K-ATPase enzyme activity in erythrocyte membranes of ACE2-inhibited SHRs after taxifolin treatment. In conclusion, ACE2 inhibition deteriorated some selected RBC properties in SHRs. Although taxifolin treatment did not improve oxidative stress markers, our data confirmed the blood pressure-lowering potential, anti-obesogenic effect, and some “erythroprotective” effects of this compound in both control and ACE2-inhibited SHRs. In vitro investigations documenting different effects of taxifolin on erythrocyte properties from control and ACE2-inhibited SHRs accentuated the irreplaceability of in vivo studies.

## 1. Introduction

Red blood cells (RBCs) are the most abundant blood elements. RBC quality plays an important role in various physiological as well as pathophysiological conditions [1]. RBCs lose the ability of protein synthesis during development, resulting in a limited possibility to repair damaged molecules, as well as their susceptibility to oxidative damage [2]. Consequently, RBC properties were suggested as suitable biomarkers of multiple diseases, and some research teams even referred to RBCs as markers of general health [3]. The ability of RBCs to repeatedly change shape according to the blood flow—the RBC deformability—represents the key ability of RBCs, which determines their survival in the circulation. However, it also represents the competence of RBCs to accomplish their primary function: oxygen transport to tissues. RBC deformability is regulated, as well as influenced, by multiple mechanisms, e.g., RBC nitric oxide (NO) production, sodium–potassium pump (Na, K-ATPase) activity, and free radical production. Given that oxidative damage occurs to a certain extent in most diseases, e.g., cardiovascular ones, it is not surprising that the RBC properties, including their deformability, are impaired in hypertension, which is one of the most common risk factors of cardiovascular diseases [4]. In blood pressure regulation and the development of hypertension, the renin–angiotensin system (RAS) plays an important role. Angiotensin converting enzyme 2 (ACE2) is responsible for the degradation of angiotensin (Ang) II to form Ang 1–7, which possess vasodilatory, antioxidative, antithrombotic, and antifibrotic effects [5,6]. The absence of a negative regulatory mechanism of RAS, which is provided by functional ACE2, leads to the acceleration of atherogenesis, responsiveness to proinflammatory stimuli [7], as well as an increase in Ang II-mediated oxidative stress [8]. It was also shown that ACE2 inhibition induced by selective inhibitor MLN-4760 resulted in worsening the erythrocyte morphology and osmotic resistance [9].

Antioxidants have been shown to be effective agents to prevent or even restore RBC properties [1]. Taxifolin (TAX), also known as dihydroquercetin or 3,5,7,3′,4′-pentahydroxyflavanone, is a polyphenol present in a wide spectrum of plant food. Various positive effects of TAX administration, including anti-inflammatory, antimicrobial, hepatoprotective, cardioprotective, or anticancer properties, were documented [10]. TAX is more water soluble when compared with other flavonoids, e.g., its derivate–quercetin, that worsened the transit through the RBC membrane [11]. Thus, TAX binds to RBCs but does not effectively accumulate there [12]. However, another study documented TAX presence in RBCs 3 weeks after of Pycnogenol^®^ treatment [13]. In addition to the antioxidant effect of TAX, its “erythroprotective” action is also exerted via the alteration of membrane properties [14]. This is noteworthy for healthy individuals whose plasma antioxidant capacity is sufficiently high and thus cannot be further increased by the addition of TAX.

The antioxidant capacity of various molecules is widely tested under in vitro conditions. However, when investigating the pathophysiology, therapeutic interventions, and complications of diverse pathologies, e.g., hypertension, animal models play an important role in studying in vivo conditions. Spontaneously hypertensive rats (SHRs) represent one of the most widely used experimental models of essential hypertension with a genetic background. In SHRs, multiple RBC abnormalities have been documented [15,16,17].

Considering the above-mentioned known facts and identifying the fields that are worthy to elucidate, we aimed to determine RBC deformability (with selected modifying factors), together with exploring the potential effects of TAX treatment in both, in vivo and in vitro conditions in SHRs, as well as in SHRs following the ACE2 inhibition.

## 2. Materials and Methods

### 2.1. Experimental Model

Males of SHRs were obtained from the accredited breeding facility: the Department of Toxicology and Laboratory Animals Breeding, Centre of Experimental Medicine, Slovak Academy of Sciences, Dobra Voda, Slovak Republic, and were kept in the certified facility at Centre of Experimental Medicine, Institute of Normal and Pathological Physiology, Slovak Academy of Sciences, Bratislava, Slovak Republic. SHRs were housed two per cage in an air-conditioned room with a 12:12 h light–dark cycle at humidity 45–65% and temperature 22–24 °C. Rats had free access to drinking water and a pelleted diet Altromin formula 1324, variant P (Altromin Spezialfutter, Lage, Germany). When the animals reached the age of 16 weeks, they were divided into four groups: control (C; *n* = 20), TAX-treated (T; *n* = 15), MLN-4760-treated (M; *n* = 20), and MLN-4760 + TAX-treated group (MT; *n* = 20). 

The specific ACE2 inhibitor MLN-4760 (MedChemExpress, Monmouth Junction, NJ, USA) was administered to experimental animals assigned to M and MT groups at a dose of 1 mg/kg/day dissolved in 10% dimethyl sulfoxide (DMSO) in isotonic saline via osmotic minipumps as described previously [9]. SHRs assigned to C and T groups were administered vehicle, i.e., 10% DMSO in isotonic saline. The systolic blood pressure (BP) and body weight (BW) of all experimental animals were registered 3 days before the osmotic minipump implantation (start value) and 1 day before the end of the experiment (end value). BP was measured by the use of tail-cuff plethysmography (MRBP, IITC Life Science Inc., LA, CA, USA). The difference between the values was calculated: Δ BP or Δ BW = end value − start value.

**Figure 1:**
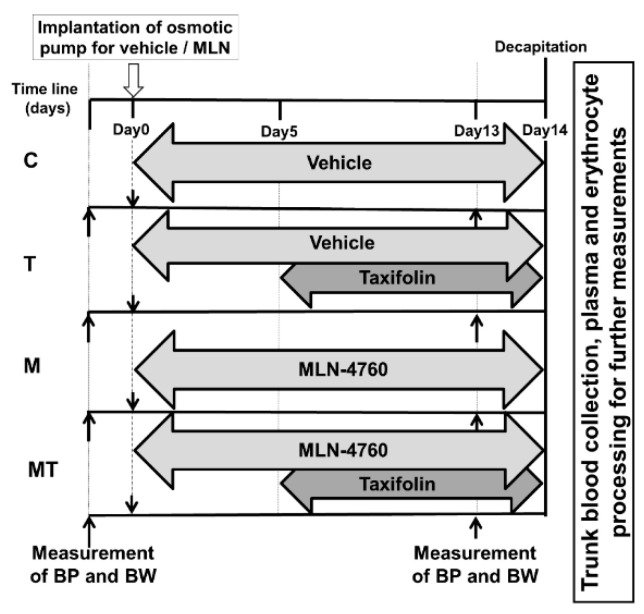
Experimental design. Abbreviations: C—control group of spontaneously hypertensive rats (SHRs), T—taxifolin-treated SHRs, M—MLN-treated (MLN-4760, ACE2 inhibitor) SHRs, MT—MLN and taxifolin-treated SHRs, BP—systolic blood pressure, BW—body weight.

Taxifolin (Cayman Chemical, Ann Arbor, MI, USA, Item No. 18647, purity 98%, batch No. 0475667-23) was dissolved in an estimated volume of tap water, which was prepared fresh every day separately for each cage of rats, and administered to rats assigned to T and MT groups at the dose 20 mg/kg/day in drinking water from the 5th day until the end of the experiment. On day 14, all the rats were exposed to brief CO_2_ anesthesia and subsequently decapitated. Trunk blood was taken into heparinized tubes (140 UI/5 mL) for further analysis from 12 animals assigned to C, M, and MT groups, and from 8 rats assigned to the T group. The experimental design is presented in Figure 1.

The basic hematologic parameters—the hematocrit value, mean cell volume (MCV), and red cell distribution width (RDW-SD)—were determined using blood analyzer Sysmex F-820 (Sysmex Corp, Tokyo, Japan). Plasma was separated by centrifugation (×850 *g*, 4 °C), and stored at −80 °C until further analyses. The buffy coat and upper 10% of RBCs were discarded. RBCs were isolated by washing them three times in saline. The part of washed RBCs (250 μL) was hemolyzed (final dilution 1:19, *v*:*v*) using cold distilled H_2_O and stored at −80 °C. To observe RBC morphology by light microscopy, 10 µl of whole blood was mixed with 90 µl of physiological solution. We quantify the occurrence of echinocytes—i.e., RBCs that lost their normal shape of biconcave disc and have multiple projections or spicules. We modified the method of Bessis et al. [18] and assigned all observed RBCs into three categories: normal discocytes, echinocytes I (irregularly contoured RBCs), and echinocytes II (RBCs with spicules). The ratio of the echinocyte count to the total count of RBCs (1300–1500 RBCs in each experimental group) was calculated. 

All experimental procedures were approved by the Ethics Committee of the Centre of Experimental Medicine, Slovak Academy of Sciences, and by the State Veterinary and Food Administration of the Slovak Republic (approval number: 2652/2021-220, date of approval: 19 March 2021), with agreement according to European Union Directive 2010/63/EU. 

This study represents a continuation of our previous study; thus, the following methods—i.e., angiotensin peptide concentration, parameters of antioxidant status and oxidative stress in blood plasma and hemolyzed RBCs, RBC deformability, NO production, free radical measurement, Na,K-ATPase enzyme kinetic method, and determination of RBC osmotic resistance, have been described in detail previously [9].

### 2.2. Angiotensin Peptide Concentration

Heparinized plasma samples were used for the quantification of angiotensin (1-10) (Ang I), angiotensin (1-8) (Ang II), angiotensin (1-7) and angiotensin (1-5) equilibrium levels by liquid chromatography mass spectrometry/mass-spectroscopy in Attoquant Diagnostics (Vienna, Austria). Derived parameters—i.e., plasma renin activity: Ang I + Ang II, ACE activity: Ang II/Ang I, and alternative RAS activity: (Ang 1-7 + Ang 1-5)/(Ang I + Ang II + Ang 1-7 + Ang 1-5)—were calculated as well. The procedures were carried out as stated previously [9].

### 2.3. Antioxidant Status and Oxidative Stress in Blood Plasma and Hemolyzed RBCs

Spectrophotometric and fluorescent analyses were performed using a Synergy H1 Hybrid Multi-mode Reader (Agilent, Santa Clara, CA, USA) and were described in detail previously [19,20]. 

As a general marker of oxidative stress, the ratio of reduced to oxidized glutathione (GSH/GSSG) was determined. As a standard, reduced L-glutathione/glutathione oxidized was used and fluorescence was measured at λ_ex_ = 350 nm and λ_em_ = 460 nm. As a marker of sample antioxidant status, the ferric-reducing antioxidant power (FRAP) was determined. The FeSO_4_*7H_2_O was used as a standard, and the absorbance was measured at λ = 593 nm. The total antioxidant capacity (TAC) with trolox and DMSO as a standard was measured at λ = 660 nm. 

The concentration of advanced glycation end products (AGEs) was determined as a marker of carbonyl stress. AGE-BSA was used as a standard, and fluorescence was measured at λ_ex_ = 370 nm and λ_em_ = 440 nm. As a marker of advanced glycation, fructosamine (FRUC) concentration was determined. As a standard, 1-deoxy-morpholino-D-fructose was used, and the absorbance was measured at λ = 530. 

Advanced oxidation protein products (AOPP) were measured as a marker of protein oxidation. Chloramine-T mixed with potassium iodide was used as a standard, and the absorbance was measured at λ = 340 nm. Thiobarbituric acid reactive substances (TBARS) measurement was used as a marker of lipid peroxidation. As the standard, 1,1,3,3-tetraethoxypropane was used, and fluorescence was measured at λ_ex_ = 515 nm and λ_em_ = 553 nm.

The GSH/GSSG ratio and FRAP were also measured in RBC hemolysates as described previously [9].

### 2.4. RBC Deformability

For the determination of RBC deformability, a filtration method was used as in our previous experiments [2,9,21]. The deformability of washed RBCs was calculated as the ratio between the RBCs that passed through the membrane filter with the pores of 5 μm in diameter (Ultrafree-MC SV Centrifugal Filter; Merck Millipore Ltd., Tullagreen Carrigtwohill, Ireland) and the RBC count before centrifugation.

### 2.5. RBC Nitric Oxide Production

To determine the RBC NO production, a fluorescent probe 4,5-diaminofluorescein diacetate (DAF-2 DA) was used as before [21]. Washed RBCs were diluted PBS and treated with DAF-2 DA. Samples were incubated in the dark at 37 °C for 10 min. The fluorescence signal was captured using filters for fluorescein isothiocyanate (λ_ex_ = 465–495 nm, λ_em_ = 515–555 nm) and a fluorescence microscope (Axio Imager M2, Zeiss, Jena, Germany). Its intensity was quantified using ZEN 3.3 Blue (Carl Zeiss Microscopy GmbH, Germany) and ImageJ 1.53e software (National Institutes of Health, Bethesda, MD, USA). RBC NO production is presented in the form of arbitrary units of a single RBC.

### 2.6. RBC Free Radical Measurement

As a fluorescent indicator of free radical production by RBCs, 2,7-dichlorofluorescein diacetate (DCF) was used as stated previously [9]. Washed RBCs were diluted in PBS and treated with DCF for 30 min in the dark at 37 °C. Subsequently, the sample was centrifuged, and the RBCs were resuspended in PBS. The same microscope, filters, and the method of quantification were used as in DAF-fluorometry.

### 2.7. Na,K-ATPase Enzyme Kinetic Method

RBC membrane isolation, as well as Na,K-ATPase kinetic measurements, were carried out as before [2,9]. Washed RBCs were homogenized in 50 mmol/L TRIS and subsequently centrifuged at 13,000× *g* for 30 min at 4 °C. The supernatant was subsequently discarded, and the sample was repeatedly homogenized and centrifuged in 30, 20 and 10 mmol/L TRIS to eliminate residual hemoglobin. Na,K-ATPase activities were measured in a range of Na^+^ concentrations (2–100 mmol/L). RBC membrane proteins (50 µg) were preincubated for 20 min at 37 °C and subsequently, ATP (final concentration 8 mmol/L, Sigma-Aldrich, St. Louis, MO, USA) was added. The chemical reaction was stopped after 20 min by 12% trichloroacetic acid. Inorganic phosphate formed by ATP hydrolysis was determined spectrophotometrically at λ = 700 nm. Measured data were used to create kinetic curves and determine the kinetic parameters of Na,K-ATPase: V_max_—maximum velocity of the enzyme reaction and K_Na_—concentration of Na^+^ required for half-maximum activation of the enzyme.

### 2.8. Determination of RBC Osmotic Resistance

RBC osmotic resistance was determined as previously [9]. A set of solutions ranging from 0% (distilled water) to 0.9% NaCl was prepared. Washed RBCs were incubated in each NaCl solution for 30 min, and the samples were centrifuged. The level of hemolysis was determined in the supernatants spectrophotometrically at λ = 540 nm. The absorbance of samples suspended in 0.9% NaCl was considered non-hemolytic, and those mixed with distilled water were used as equivalent to 100% hemolysis. Subsequently, the concentration of NaCl resulting in 50% hemolysis (IC_50_) was calculated. A decrease in IC_50_ value corresponds to an enhanced RBC osmotic resistance.

### 2.9. In Vitro Study

As the aim of the present study was also to describe the TAX-induced changes of RBC properties in in vitro conditions, washed RBCs were incubated in PBS with TAX (100 µmol/L dissolved in DMSO) at 37 °C for 1 h. For comparison, control RBCs were incubated in the same way with the vehicle. Following the incubation, the suspension was centrifuged, the supernatant was discarded, and RBCs were provided for measurements of RBC deformability, NO production, free radical measurement, and osmotic resistance as described above (Section 2.4, Section 2.5, Section 2.6 and Section 2.8).

### 2.10. Statistical Analyses

Outliers were detected using the standardized Grubbs test and removed from further analyses. The normality of the data was analyzed by D’Agostino–Pearson test. Data are presented as means ± standard deviations or as medians with interquartile ranges in case of non-Gaussian distribution. Statistical significance was analyzed by two-way ANOVA with main factors: MLN-4760 administration (i.e., ACE2 inhibition) and TAX treatment, followed by Sidak’s multiple comparison test. For non-parametric data, we worked with logarithmically transformed data to induce the normal distribution appropriate for ANOVA analysis. Differences were considered significant at *p* < 0.05. Software GraphPad Prism 8.2.1 (GraphPad Software, San Diego, CA, USA) was used for data analysis.

## 3. Results

### 3.1. Basic Biometric Parameters

The measured data with the statistical evaluation are presented in Table 1. Systolic BP of rats in individual groups were (in mmHg): 160.8 ± 24.4—start, 170.1 ± 27.9—end in C; 163.8 ± 5.5—start, 156.8 ± 9.9—end in T; 162.6 ± 23.5—start, 174.2 ± 30.6—end in M; 165.3 ± 20.1—start, 177.3 ± 26.9—end in MT group. Regarding changes in the systolic BP values, TAX treatment (*p* = 0.038; F_(1,63)_ = 4.499), MLN-4760 administration (*p* = 0.006; F _(1,63)_ = 8.017), as well as their interaction (*p* = 0.03; F_(1,63)_ = 8.017), were evaluated as statistically significant. MLN-4760 administration resulted in a higher BP increase independently of TAX treatment. However, only C and T groups differed significantly in multiple comparison test (*p* = 0.009). 

Concerning the BW changes, TAX treatment (*p* < 0.0001; F_(1,71)_ = 18.04), MLN-4760 administration (*p* = 0.025; F_(1,71)_ = 5.264), together with their interaction (*p* = 0.019; F_(1,71)_ = 5.721), was significant as well. Rats in the M group had significantly higher BW gain in comparison with the C group (*p* = 0.002), and TAX treatment in ACE2-inhibited rats abolished this effect (M versus MT, *p* < 0.0001). TAX treatment was also a significant factor for the ratio liver weight/tibia (*p* = 0.015; F_(1,39)_ = 6.449): the normalized liver weight was lower in TAX-treated SHRs independently of MLN-4760 administration. Hematocrit value, as well as the normalized weight of the kidney and heart, was not affected by any given medication.

### 3.2. Angiotensin Peptide Concentration

Plasma levels of the selected angiotensin peptides, as well as derived parameters, are presented in Table 2. TAX treatment did not influence any of the determined RAS components. ACE2 inhibition by MLN-4760 was a significant factor for the Ang II concentration (*p* = 0.004; F_(1,23)_ = 10.58) as well as for PRA (*p* = 0.021; F_(1,23)_ = 6.156). Both were increased in SHRs following the MLN-4760 administration independently of TAX treatment, however, without significant differences among groups in the multiple comparison test.

### 3.3. Parameters of Antioxidant Status and Oxidative Stress in Blood Plasma and Hemolyzed RBCs

Focusing on the blood plasma, a statistical analysis of the GSH/GSSG ratio revealed the factors TAX (*p* = 0.025; F_(1,34)_ = 5.490), and MLN (*p* = 0.017; F_(1,34)_ = 6.304) as statistically significant. SHRs assigned to the M group had a significantly higher GSH/GSSG plasma ratio in comparison with the C group (*p* = 0.001), and TAX treatment abolished this effect (M versus MT, *p* = 0.006). For FRUC concentration, the significance was observed only for the TAX versus MLN interaction (*p* = 0.017, F_(1, 37)_ = 6.236). For TBARS, the TAX factor (*p* = 0.014; F_(1,40)_ = 6.548) was statistically significant: TBARS concentrations were higher in TAX-treated SHRs independently of MLN-4760 administration. SHRs assigned to the MT group had higher plasma TBARS levels when compared with those in the M group (*p* = 0.03) in a multiple comparison test. There were no additional statistically significant differences among other determined parameters of antioxidant status and oxidative stress in blood plasma. A summary of the values, as well as statistical analysis, is available in Table 3.

Focusing on the hemolysates, the factor TAX (*p* = 0.003; F_(1,37)_ = 9.920), as well as the interaction between TAX and MLN (*p* = 0.006; F_(1,37)_ = 8.645), were significant for the GSH/GSSG ratio. SHRs assigned to the C group had a higher GSH/GSSG ratio in comparison with the T group (*p* = 0.001), as well as with the M group (*p* = 0.019). Regarding FRAP levels in hemolysates, a two-way ANOVA revealed the interaction of both factors, TAX and MLN, as significant (*p* = 0.019; F_(1,39)_ = 5.962). TAX treatment led to a decrease in FRAP levels only in ACE2-inhibited SHRs (M versus MT, *p* = 0.046) (Table 4).

### 3.4. Erythrocyte Parameters MCV and RDW-SD

**Figure 2:**
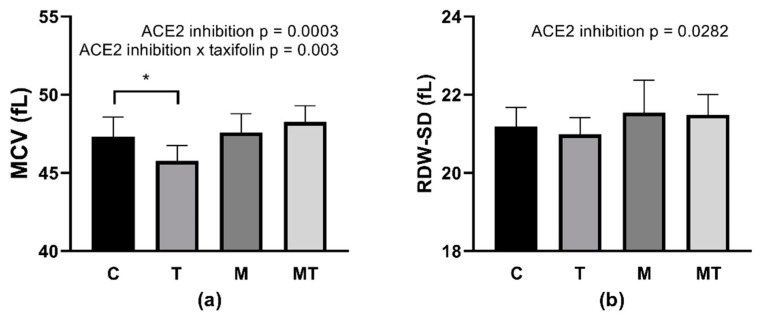
Erythrocyte parameters MCV (**a**), RDW-SD (**b**). Abbreviations: MCV—mean cell volume, RDW-SD—red cell distribution width, C—control group (*n* = 11–12), T—taxifolin-treated group (*n* = 8), M—MLN-treated (MLN-4760, ACE2 inhibitor) group (*n* = 12), MT—MLN- and taxifolin treated group (*n* = 12). Data are presented as mean ± standard deviations. Statistical significance: * *p* < 0.05.

For the MCV value, a two-way ANOVA revealed factor MLN (*p* = 0.0003; F_(1,40)_ = 15.71) as well as the interaction of MLN and TAX factors (*p* = 0.003; F_(1,40)_ = 9.983) statistically significant (Figure 2a). Control SHRs treated with TAX had lower MCV in comparison with non-treated animals (*p* = 0.011). 

Systemic ACE2 inhibition resulted in the significantly increased RDW-SD independently of TAX treatment (*p* = 0.0282; F_(1,39)_ = 5.198), however, without differences among groups in the multiple comparison test (Figure 2b).

### 3.5. RBC Deformability, Osmotic Resistance, RBC Free Radical, and NO Production

**Figure 3:**
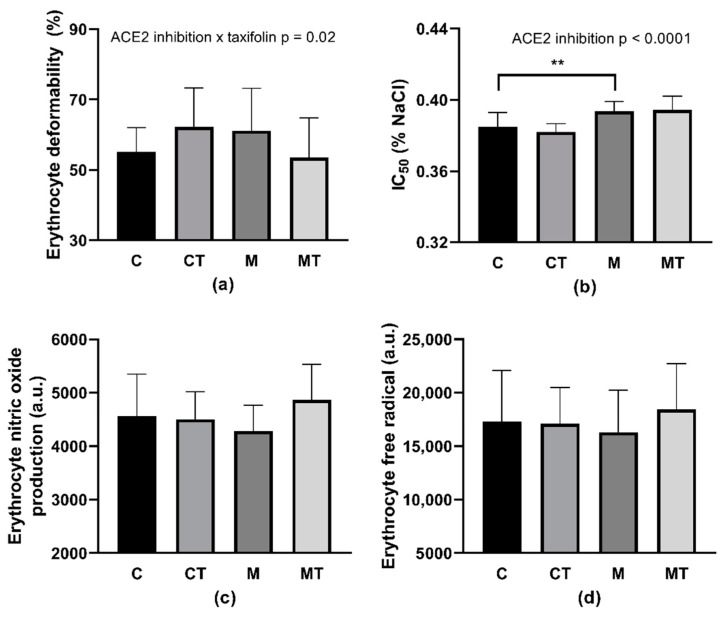
Erythrocyte deformability (**a**), osmotic resistance (IC_50_) (**b**), nitric oxide produced by erythrocytes (**c**), and free radical production in erythrocytes (**d**). Abbreviations: C—control group (*n* = 11–12), T—taxifolin-treated group (*n* = 7–8), M—MLN-treated (MLN-4760, ACE2 inhibitor) group (*n* = 12), MT—MLN- and taxifolin-treated group (*n* = 10–12), IC_50_–NaCl concentration at which 50% hemolysis occurred, a.u.–arbitrary units. Data are presented as mean ± standard deviations. Statistical significance: ** *p* < 0.01.

An evaluation of RBC deformability by the two-way ANOVA revealed the interaction ACE2 inhibition × TAX (*p* = 0.0272; F_(1,40)_ = 5.26) as significant but without any difference among groups (Figure 3a) in multiple comparisons. Regarding IC_50_ value, ACE2 inhibition was statistically significant (*p* < 0.0001; F_(1,39)_ = 25.33): MLN-4760 administration increased the IC_50_ value that corresponds to the deterioration of RBC osmotic resistance following the ACE2 inhibition independently of TAX treatment (Figure 3b). Rats assigned to the M group had lower osmotic resistance in comparison with those in the C group (*p* = 0.006). NO production by RBCs was not affected by any of the given medication. However, the difference in RBC NO production between the M and the MT groups with a *p*-value of 0.059 was noted as closest to statistical significance (Figure 3c). Neither ACE2 inhibition nor TAX treatment affected DCF-related fluorescence (Figure 3d). 

### 3.6. Na,K-ATPase Enzyme Kinetics

**Figure 4:**
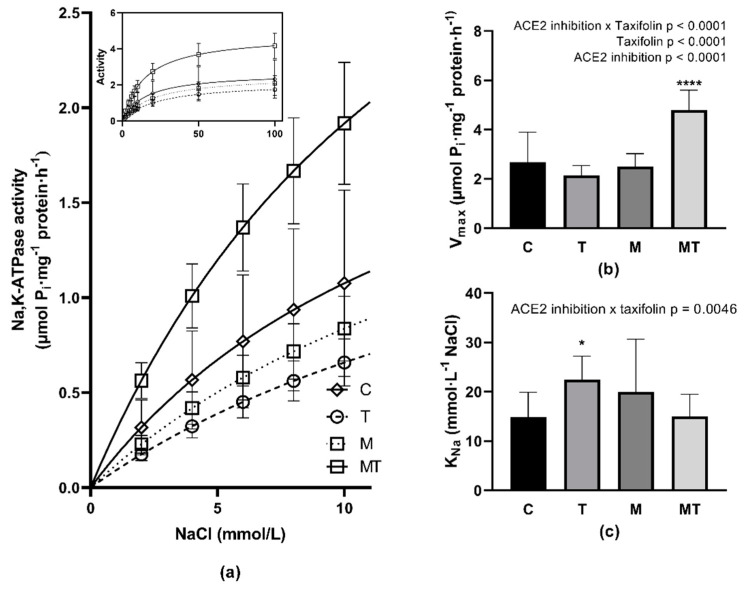
Activation of the Na,K-ATPase in Na^+^ concentrations ranging from 2 to 10 mmol/L; insert—activation of the enzyme in the whole investigated concentration range of NaCl (**a**), kinetic parameters of Na,K-ATPase V_max_ (**b**) and K_Na_ (**c**) in erythrocyte membranes. Abbreviations: C—control group (*n* = 15), T—TAX-treated group (*n* = 9), M—MLN-treated (MLN-4760, ACE2 inhibitor) group (*n* = 18), MT—TAX and MLN-treated group (*n* = 15). V_max_—maximal velocity of reaction. K_Na_—NaCl concentration required for ½ maximal activation of Na,K-ATPase, data are presented as means ± standard deviations. Statistical significance: * *p* < 0.05 versus C group, **** *p* < 0.0001 versus M group.

Inhibition of ACE2 induced by the MLN-4760 administration decreased Na,K-ATPase activity in RBC membranes, particularly in the concentration range of sodium, which corresponds with its physiologically relevant intracellular concentrations (Figure 4a). However, the kinetic evaluation of obtained data resulted in no difference regarding the ability of Na,K-ATPase enzyme to bind Na^+^ ions, as an increase in the K_Na_ value was statistically insignificant (C versus M, *p* = 0.088, Figure 4c). The number of active Na,K-ATPase molecules also remained stable as indicated by the unchanged V_max_ value between the C and M groups (Figure 4b).

The treatment of control animals with TAX resulted in lowered Na,K-ATPase in RBC membranes, particularly at physiologically relevant intracellular concentrations of sodium (Figure 4a). The kinetic evaluation of obtained data resulted in a significantly decreased ability of Na,K-ATPase enzyme to bind Na^+^ ions as shown by a statistically significant increase in the K_Na_ value (C versus T, *p* = 0.029, Figure 4c). The number of active Na,K-ATPase molecules was not altered as indicated by similar V_max_ values between the C and T groups. TAX treatment of ACE2-inhibited animals was followed by a significant increase in the Na,K-ATPase activity in RBCs, particularly in the concentration range of sodium corresponding to its physiologically relevant intracellular concentrations. The observed TAX-induced stimulation of the enzyme in ACE2-inhibited animals was most probably caused by a higher number of active Na,K-ATPase molecules as suggested by increased V_max_ value (M versus MT, *p* < 0.0001, Figure 4b).

### 3.7. Erythrocyte Morphology

**Figure 5:**
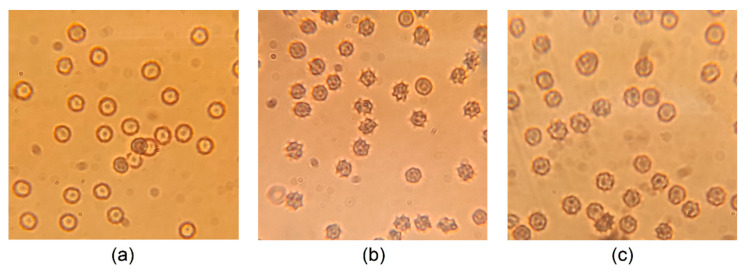
Representative photographs of erythrocyte morphology. Control group (**a**), MLN-4760-treated group (ACE2 inhibition) (**b**), MLN-4760 and taxifolin-treated group (**c**).

In MLN-4760-treated rats, almost 60% of experimental animals had abnormal RBCs (echinocytes I and echinocytes II). Abnormalities in the shape were also present in RBCs of SHRs assigned to the MT group, however, they were less frequent and intense in comparison with the M group. Quantitative analysis is presented in Table 5, and representative photographs are shown in Figure 5. In this analysis, we did not work with RBCs taken from the T group.

### 3.8. RBC Deformability, Osmotic Resistance, RBC Free Radical, and NO Production after Incubation with TAX In Vitro

**Figure 6:**
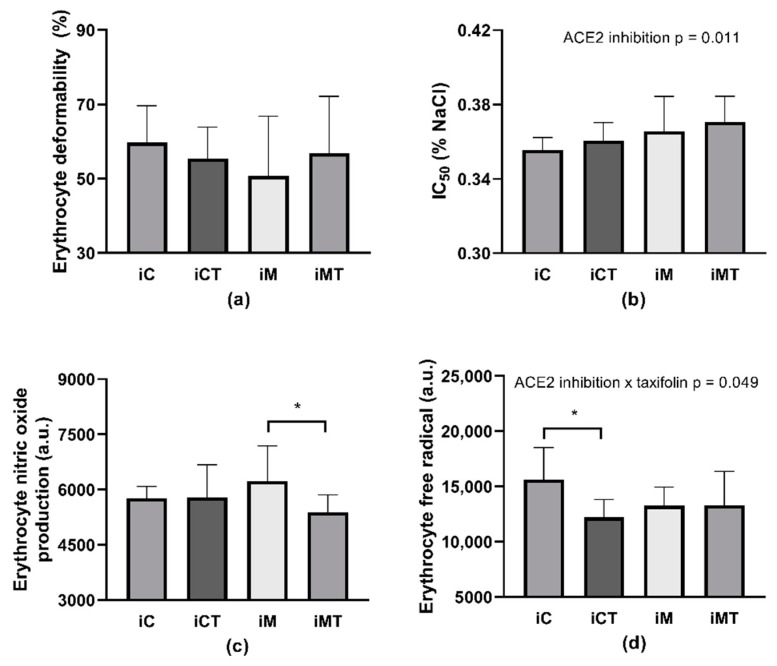
Erythrocyte deformability (**a**), osmotic resistance (IC50) (**b**), nitric oxide produced by erythrocytes (**c**), and free radical production in erythrocytes (**d**) after in vitro taxifolin treatment. Abbreviations: iC—erythrocytes of control rats incubated with vehicle, iCT—erythrocytes of control rats incubated with taxifolin, iM—erythrocytes of MLN-treated rats (MLN-4760, ACE2 inhibitor) incubated with vehicle, iMT—erythrocytes of MLN-treated rats incubated with taxifolin, a.u.—arbitrary units. Data are presented as means ± standard deviations. Statistical significance: * *p* < 0.05. The count of samples per group: *n* = 6 for RBC deformability; *n* = 11–12 for osmotic resistance; iC and iCT *n* = 5–6, iM and iMT *n* = 9–10 for other parameters.

Considering the in vitro part of the experiment, there were no changes in the deformability of RBCs taken from control SHRs as well as MLN-4760-treated ones, following the incubation with TAX (Figure 6a). Regarding the RBC osmotic resistance, similarly to the in vivo experiment, only ACE2 inhibition was significant (*p* = 0.011; F_(1,42)_ = 7.057). RBCs taken from MLN-4760-treated SHRs had worsened osmotic resistance regardless of incubation with TAX (Figure 6b).

The incubation of RBCs from ACE2-inhibited SHRs with TAX lowered RBC NO production (*p* = 0.044, Figure 6c). Regarding the free radical production in RBCs, TAX and ACE2 inhibition interaction was significant (*p* = 0.049; F_(1,28)_ = 4.218). RBCs taken from control SHRs had lower free radical production after incubation with TAX in comparison with RBCs incubated with vehicle (*p* = 0.031). This effect of TAX was not observed in RBCs taken from ACE2-inhibited SHRs (Figure 6d).

## 4. Discussion

It was shown that antioxidants may prevent the deterioration of RBC properties in hypertension. We presumed that RBC quality might be further worsened in hypertensives in the condition of ACE2 inhibition. Therefore, we aimed to characterize changes induced by TAX treatment in SHRs with a focus on RBC characteristics.

### 4.1. Basic Biometric Parameters and Angiotensin Peptides Concentration

Basic biometric parameters, as well as concentrations of angiotensin peptides, were measured in order to determine the condition of all experimental animals with emphasis on the main effects of systemic ACE2 inhibition and TAX treatment. The increase in BW gain following the ACE2 inhibition observed in this experiment is consistent with data showing a BW decrease after the administration of an ACE2 activator [22] as well as the documented negative correlation between circulating ACE2 levels and BW in rats [23]. We observed a decrease in BW gain following the TAX treatment mainly in ACE2-inhibited SHRs to values comparable to TAX-treated control SHRs. A similar decrease in BW gain after the TAX administration was observed in mice fed a high-fat diet [24], despite no effect on food and water intake being noticed. Although BW gain was not modified by TAX administration in our control SHRs, TAX at lower doses—i.e., 100 and 300 μg/kg/day reduced BW gain in SHRs with age [25]. The decrease in normalized liver weight after TAX treatment could be related to the reported hepatoprotective effects of TAX [10], as liver weight was shown to be significantly higher in SHRs than in normotensive controls regardless of diet [26].

The more intense BP rise in SHRs after the ACE2 inhibition (significance in MLN factor) was expected and could be related to the higher Ang II concentration observed in these experimental animals. Regarding the antihypertensive effect of TAX, it has been discussed, however, that the available studies are quite contradictory. According to Plotnikov et al. [27], TAX treatment (20 mg/kg/day for 6 weeks) led to a decrease in the mean BP, whole blood viscosity, as well as to the promotion of vasodilation of the thoracic aorta in SHRs. An antihypertensive effect of TAX (at two doses–25 and 50 mg/kg) was also observed in SHRs fed by fructose [28]. In contrast, the BP-lowering potential of TAX was not confirmed by other studies [25,29]. What could interfere with the resulting effect of TAX on BP, e.g., dose of TAX, vehicle, way of administration, comorbidities, will have to be clarified in the future. In our study, TAX treatment lowered the BP rise mainly in control SHRs, not in the ACE2-inhibited animals. Thus, TAX-induced changes in BP could be at least partially explained due to the effect of TAX on RAS components. Alterations in the concentration or activity of RAS peptides following the TAX administration were documented previously [25,30]; however, even in this case, the recognized TAX effect is not entirely consistent and is probably dose-dependent [25]. Although we observed a different response of control and ACE2-inhibited SHRs to TAX administration in terms of BP values, no significant changes in RAS peptide concentrations after TAX administration were noticed in this study.

The treatment with MLN-4760 inhibitor led to an increase in plasma Ang II concentration and calculated plasma renin activity as expected, without changes in other observed RAS components in blood plasma. It should be also noted that despite the selectivity of MLN-4760, it does not inhibit ACE2 entirely [31]. Furthermore, the effect could be more observable at the level of local RAS; e.g., ACE2 inhibition resulted in a remarkable elevation of local Ang II production in the aortas of diabetic mice, although Ang II levels in serum were unaltered [32]. In the renal tissue, MLN-4760 selectively blocked Ang 1-7 formation at low Ang II concentrations; however, Ang 1-7 levels seem to be unaffected at higher Ang II concentrations [33].

As it was shown that hypertension, as well as the modulation of RAS peptides, could be related to an increase in free radical production, we were also focused on oxidative stress markers in our experimental animals [1,8].

### 4.2. Markers of Oxidative Stress and Antioxidant Status in Plasma and Hemolysates

It could be presumed that ACE2 inhibition leads to an increase in Ang II levels and consequently to Ang II-induced oxidative stress. However, in our experiment, MLN-4760 administration elevated the GSH/GSSG ratio in blood plasma, suggesting some systemic adaptive responses in SHRs after ACE2 inhibition. In contrast, the GSH/GSSH ratio was lower in hemolysates of ACE2-inhibited SHRs. One may speculate that it could be related to the disturbance of the oxidative balance in the intracellular compartment following MLN-4760 administration in SHRs.

The properties of TAX to mitigate the oxidative and carbonyl stress are well known [34,35]; however, a variety of studies tested TAX as a part of plant extracts, i.e., in combination with other flavonoids that may exert a synergistic effect. In our study, we have not observed significant antioxidant action of TAX when focusing on markers in blood plasma. Instead, TAX treatment surprisingly increased plasma TBARS levels significantly in ACE2-inhibited SHRs. An increased GSH/GSSG ratio in ACE2-inhibited SHRs was normalized to values observed in control rats after TAX administration.

### 4.3. Characteristics of Erythrocytes

As RBC properties are fundamentally affected by oxidative stress, which is deeply connected to Ang II effect and hypertension [1,36], experiments with TAX administration, which might affect any of them, could shed new light on their relationship. After TAX treatment, sixteen TAX metabolites were detected with varying concentrations in plasma, tissues, urine, and feces [37]. Therefore, a different effect of TAX administered per os and in vivo might be present, however, comparison studies of these approaches, especially with an emphasis on RBC characteristics, are limited. Thus, in this study, we focused on various RBC parameters, under monitoring the oxidative stress markers in plasma and hemolysates, together with RAS peptides following the ACE2 inhibition and TAX treatment during in vivo and in vitro conditions in SHRs.

Multiple RBC abnormalities have been already observed in hypertensive individuals, including SHRs [4]. Nevertheless, little is known regarding the consequences of systemic ACE2 inhibition on RBC quality. The physiological shape of RBCs is a biconcave discoid shape—i.e., RBCs are also referred to as discocytes. It was shown that discocytes can transform into echinocytes under certain conditions. Echinocytes may occur as artefacts if glass slides are used during blood smear preparation; however, echinocyte transformation can be a consequence of various intrinsic and extrinsic factors [18]. As the sample handling was always the same, one can presume that an increase in echinocyte occurrence can be related to the worsened ability of RBCs to keep the shape of the biconcave disc. Thus, this experiment documented the MLN-4760-induced deterioration of RBC morphology (by the use of light microscope) as well as osmotic resistance. A test of RBC osmotic stability is commonly used in studies of RBC membrane permeability. In addition, RBC osmotic resistance could serve as a potential biomarker of oxidative membrane damage in various pathologic conditions [38]. The evaluation of RBC osmotic resistance in ACE2-inhibited animals indicated that 50% hemolysis occurred at higher NaCl concentration than in control SHRs—i.e., ACE2 inhibition worsened RBC osmotic stability. Another RBC parameter, RDW, represents a measure of the change in RBC size. Abnormalities in RDW value could help for differential diagnosis of anemia types. In addition, a relationship between an increase in RDW value and the risk of death in general (most prominently for CVD diseases) was also observed [39,40,41]. Therefore, an increase in the RDW value could predict a worse prognosis in ACE2-inhibited SHRs [42]. As the presence of ACE2 was confirmed only in RBC progenitors, not in matured RBCs [43], the effect of short-term MLN-4760 administration (2 weeks lasting) on RBC properties seems to be indirect.

Focusing on the effect of TAX treatment, our data could extend the knowledge from previous research on flavonoids and RBC properties. TAX administration resulted in a reduction in MCV which is a measure reflecting the RBC volume, however, only in control SHRs. In humans, a higher MCV value was documented, e.g., in patients with nutritional deficiencies, certain medications, and bone-marrow disorders [44]. Statistical analysis of routine MCV and RDW measurements was shown to effectively predict acute mortality in patients after their treatment in the emergency department. Although we did not notice any difference in RDW following the TAX treatment, the decrease in MCV may be related to its beneficial effect, however, neutralized by ACE2 inhibition. The study focused on the action of quercetin documented even more significant changes in MCV after incubation of RBC with quercetin [45]. Nevertheless, we were not able to detect the TAX-induced enhancement of RBC osmotic resistance that was documented after the quercetin administration [45,46]. However, our experiments were conducted using SHRs, and a higher osmotic resistance was already confirmed in this rat strain in comparison with control normotensive Wistar–Kyoto rats [15]. One may speculate that there is no reserve for further improvement of RBC osmotic resistance.

TAX treatment was shown to improve whole blood viscosity when administered alone [27], as well as a part of ascovertin, a complex flavonoid of TAX with ascorbic acid [47]. In the latter study, an improvement of RBC aggregability and deformability was also observed but not when patients were treated by TAX alone. This effect may be explained by the effect of ascorbic acid [2]. TAX treatment was not efficient to improve RBC deformability in our study as well, and similarly, no effects were observed regarding the NO and free radical production by RBCs during in vivo conditions. Studies with a longer period of TAX administration or other doses of TAX could reveal its beneficial effects on RBCs in the future.

The quality of RBCs is largely dependent on the activity of the Na,K-ATPase enzyme that is localized in membranes. This active transport mechanism is necessary to maintain the normal intracellular ion concentration with a significant impact on RBC deformability. A positive effect of TAX on renal Na,K-ATPase activity in SHRs with metabolic syndrome was documented [28]. Although the effect of TAX on Na,K-ATPase in RBCs was not documented yet, other related flavonoids—e.g., quercetin—were proven to increase human RBC Na,K-ATPase activity in vitro [48] or in vivo [49]. The most significant effect of TAX administration on Na,K-ATPase activity was observed in ACE2-inhibited SHRs, in which the enzyme was clearly able to benefit from TAX administration over a wide range of sodium ion concentrations. Regarding the Na,K-ATPase activity in RBCs of control SHRs, the enzyme showed a decrease in the ability to bind sodium ions—as indicated by an increase in K_Na_ value—however, without significant consequences on RBC deformability. The possible mechanisms responsible for Na,K-ATPase alterations could involve the direct antioxidant effect of TAX or an ability of TAX to modulate RBC membrane quality in an antioxidant action-independent mechanism [14].

The in vitro part of this study documented different effects of TAX treatment on RBC properties in control and ACE2-inhibited SHRs in comparison with TAX action observed in vivo. An incubation of RBCs with TAX decreased DCF-related fluorescence that is corresponding to free radical production only in control SHRs. It is noteworthy that such an antioxidant action of TAX was not documented in RBCs during in vivo study. RBCs taken from ACE2-inhibited animals did not change DCF-related fluorescence—a marker of oxidative stress—following the TAX treatment neither in vivo nor in vitro. Regarding RBC NO production, TAX administration in vitro seems to affect only ACE2-inhibited rats, in which it led to a decrease in NO production. Regarding the in vitro study, this was a pilot experiment that aimed to check whether the effect of TAX on erythrocytes is the same as during in vivo conditions or not. As we noticed that in vitro response is not consistent with the in vivo effect, this issue needs to be clarified in future studies (e.g., using a different dose, duration of incubation).

## 5. Conclusions

Although the ACE2 presence has not been documented in RBC membranes, the systemic inhibition of ACE2 by MLN-4760 deteriorated some RBC properties in SHRs. Regarding the TAX treatment, we have not noticed unequivocal changes in selected oxidative stress markers in blood plasma confirming the antioxidant action of TAX. However, our data confirmed the blood pressure-lowering potential, anti-obesogenic effect, and some “erythroprotective” effects of TAX in control SHRs, as well as in ACE2-inhibited ones.

## Figures and Tables

**Table 1:** Basic biometric parameters.

Parameter	Experimental Groups				2-Way ANOVA		
	C	T	M	MT	TAX	MLN	Inter.
Δ BP (mmHg)	9.28 ± 15.25	−7 ± 10.56 ^††^	11.56 ± 18.66	11.94 ± 13.97	x	xx	x
Δ Body weight (g)	17.7 ± 5.8	14.8 ± 8.7	24.9 ± 6.2 ^††^	14.7 ± 6 ****	xxxx	x	x
HW/Tibia (mg/mm)	33.3 ± 3.6	33.1 ± 1.8	33.9 ± 2.1	32.1 ± 1.3			
LW/Tibia (mg/mm)	344 ± 31	325 ± 20	353 ± 30	330 ± 22	x		
KW/Tibia (mg/mm)	64.4 ± 2.7	62.9 ± 3.3	64.9 ± 3.9	63.5 ± 1.6			
Hematocrit (%)	50 ± 3.2	52 ± 2	51.3 ± 1.6	50.9 ± 0.5			

Abbreviations: BP—blood pressure, HW—heart weight, LW—liver weight, KW—kidney weight, C—control group, T —TAX-treated group, M—MLN-treated (MLN-4760, ACE2 inhibitor) group, MT— MLN and taxifolin-treated group, TAX—taxofolin administration, MLN—MLN administration, Inter.—interaction between TAX and MLN factors. Data are presented as mean ± standard deviations. Statistical significance: ^††^ *p* < 0.01 vs. C; **** *p* < 0.0001 vs. M; x *p* < 0.05, xx *p* < 0.01, xxxx *p* < 0.0001 for the corresponding factors and their interaction. The count of animals per group: C, M, and MT groups—*n* = 20, T group—*n* = 15; for hematocrit value: C, M, and MT groups—*n* = 12, T group—*n* = 8.

**Table 2:** Angiotensin Peptide Concentration in Blood Plasma.

Parameter	Experimental Groups				2-Way ANOVA		
	C	T	M	MT	TAX	MLN	Inter.
Ang I (1-10) (pmol/L)	94.5 ± 17	93.3 ± 32.2	106 ± 25	120 ± 26			
Ang II (1-8) (pmol/L)	161 ± 32	137 ±11	186 ± 35	192 ± 36		xx	
Ang 1-7 (pmol/L)	6.6 ± 2.59	5.5 ± 1.7	6.36 ± 2.34	6.8 ± 1.9			
Ang 1-5 (pmol/L)	20.3 ± 3.3	18.8 ± 4.7	20.9 ± 5.5	23.5 ± 3.1			
PRA (pmol/L)	255 ± 45	241 ± 61	292 ± 57	311 ± 57		x	
ACE	1.72 ± 0.25	1.65 ± 0.25	1.79 ± 0.25	1.62 ± 0.23			
ALT	0.096 ± 0.011	0.091 ± 0.008	0.084 ± 0.01	0.089 ± 0.008			

Abbreviations: Ang—angiotensin, PRA—plasma renin activity = Ang I + Ang II, ACE—angiotensin converting enzyme activity = Ang II/Ang I, ALT—alternative renin-angiotensin system activity = (Ang 1-7 + Ang 1-5)/(Ang I + Ang II + Ang 1-7 + Ang 1-5), C—control group (*n* = 7), T—taxifolin-treated group (*n* = 5), M—MLN-treated (MLN-4760, ACE2 inhibitor) group (*n* = 7), MT—MLN and taxifolin-treated group (*n* = 8), TAX—taxifolin administration, MLN—MLN administration, Inter.—interaction between TAX and MLN factors. Data are presented as mean ± standard deviations. Statistical significance: x *p* < 0.05, and xx *p* < 0.01 for the corresponding factor.

**Table 3:** Parameters of Antioxidant Status and Oxidative Stress in Blood Plasma.

Parameter	Experimental Groups				2-Way ANOVA		
	C	T	M	MT	TAX	MLN	Inter.
GSH/GSSG	7.05 (5.66; 12.4)	7.13 (5.06; 12.2)	23.9 (10.5; 35,5) ^††^	8.77 (7.22; 10.8) **	x	x	
FRAP (μmol/L)	520 ± 101	499 ± 73.5	465 ± 58.1	494 ± 77.9			
TAC (μmol/L)	1.83 ± 0.21	1.88 ± 0.27	1.71 ± 0.19	1.86 ± 0.17			
AGEs (g/g protein)	0.06 ± 0.01	0.07 ± 0.01	0.069 ± 0.02	0.066 ± 0.01			
FRUC (mmol/g protein)	0.09 ± 0.02	0.12 ± 0.05	0.10 ± 0.03	0.087 ± 0.01			x
AOPP (μmol/g protein)	13.15 ± 5.11	13.4 ± 6.52	13.1 ± 4.22	10.56 ± 2.55			
TBARS (μmol/L)	410 ± 129	489 ± 160	350 ± 83	505 ± 202 *	x		

Abbreviations: GSH—reduced glutathione, GSSG—oxidized glutathione, FRAP—ferric-reducing antioxidant power, TAC—total antioxidant capacity, AGEs—advanced glycation end products, FRUC—fructosamine, AOPP—advanced oxidation protein products, TBARS—thiobarbituric acid reactive substances, C—control group (*n* = 11–12), T—taxifolin-treated group (*n* = 7–8), M—MLN-treated (MLN-4760, ACE2 inhibitor) group (*n* = 11–12), MT—MLN and taxifolin-treated group (*n* = 11–12), TAX—taxifolin administration, MLN—MLN administration, Inter.—interaction between TAX and MLN factors. Data are presented as mean ± standard deviation for parametric data or as median and interquartile range for non-parametric data. Statistical significance: ^††^ *p* < 0.01 vs. C; * *p* < 0.05, and ** *p* < 0.01 vs. M; x *p* < 0.05 for the corresponding factors and their interaction.

**Table 4:** GSH/GSSG Ratio and FRAP Concentration in the Hemolysates.

Parameter	Experimental Groups				2-Way ANOVA		
	C	T	M	MT	TAX.	MLN	Inter.
GSH/GSSG	0.37 ± 0.222	0.10 ± 0.025 ^†††^	0.22 ± 0.1 ^†^	0.21 ± 0.08	xx		xx
FRAP (mmol/L)	13.0 ± 1.94	14.5 ± 3.87	15.4 ± 2.26	12.7 ± 3.03 *			x

Abbreviations: GSH—reduced glutathione, GSSG—oxidized glutathione, FRAP—ferric-reducing antioxidant power, C—control group (*n* = 11), T—taxifolin-treated group (*n* = 7–8), M—MLN-treated (MLN-4760, ACE2 inhibitor) group (*n* = 12), MT—MLN and taxifolin-treated group (*n* = 11–12), TAX—taxifolin administration, MLN–MLN administration, Inter.–interaction between TAX and MLN factors. Data are presented as the mean ± standard deviation. Statistical significance: ^†^ *p* < 0.05, and ^†††^ *p* < 0.001 vs. C; * *p* < 0.05 vs. M; x *p* < 0.05, and xx *p* < 0.01 for the corresponding factors and their interaction.

**Table 5:** Portions of individual RBC shapes in peripheral blood smears.

Erythrocyte Shape	Experimental Groups		
	C	M	MT
Normal	88.6%	40.5%	60%
Echinocyte I	11.4%	51.8%	35.5%
Echinocyte II	0%	7.7%	4.5%

Abbreviations: C—control group (*n* = 8), M—MLN-treated (MLN-4760, ACE2 inhibitor) group (*n* = 6), MT—MLN- and taxifolin-treated group (*n* = 8). Data are presented as the percentage of normal erythrocytes (shape of biconcave disc) or echinocytes (echinocytes I—irregularly contoured erythrocytes, echinocytes II—erythrocytes with spicules) among the total erythrocyte count (1300–1500 erythrocytes in each experimental group).

## Data Availability

The data that support the findings of this study are available in this article or from the corresponding author upon reasonable request.
